# Tris(3-amino­pyrazine-2-carboxyl­ato-κ^2^ *N* ^1^,*O*)diaqua­erbium(III) tetra­hydrate

**DOI:** 10.1107/S1600536811034398

**Published:** 2011-08-27

**Authors:** Shan Gao, Seik Weng Ng

**Affiliations:** a Key Laboratory of Functional Inorganic Materials Chemistry, Ministry of Education, Heilongjiang University, Harbin 150080, People’s Republic of China; b Department of Chemistry, University of Malaya, 50603 Kuala Lumpur, Malaysia; c Chemistry Department, Faculty of Science, King Abdulaziz University, PO Box 80203, Jeddah, Saudi Arabia

## Abstract

The water-coordinated Er^III^ atom in the title compound, [Er(C_5_H_4_N_3_O_2_)_3_(H_2_O)_2_]·4H_2_O, is *N*,*O*-chelated by three 3-amino­pyrazine-2-carboxyl­ate ions and has a square-anti­prismatic geometry. The mononuclear mol­ecule inter­acts with the solvent water mol­ecules to generate a three-dimensional hydrogen-bonded network.

## Related literature

For a related structure, see: Leciejewicz *et al.* (2004 ▶).
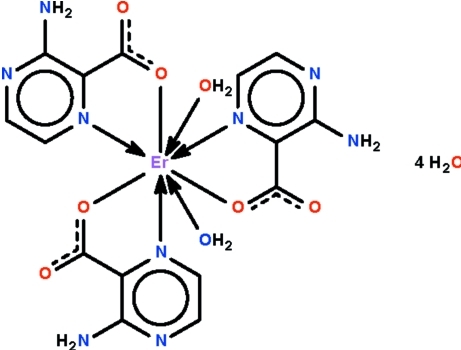


## Experimental

### 

#### Crystal data

[Er(C_5_H_4_N_3_O_2_)_3_(H_2_O)_2_]·4H_2_O
*M* *_r_* = 689.69
Monoclinic, 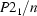

*a* = 9.1915 (9) Å
*b* = 19.7152 (14) Å
*c* = 13.5637 (11) Å
β = 105.276 (3)°
*V* = 2371.1 (3) Å^3^
*Z* = 4
Mo *K*α radiation
μ = 3.62 mm^−1^
*T* = 291 K
0.25 × 0.20 × 0.15 mm

#### Data collection

Rigaku R-AXIS RAPID IP diffractometer
Absorption correction: multi-scan (*ABSCOR*; Higashi, 1995 ▶) *T*_min_ = 0.465, *T*_max_ = 0.613
18438 measured reflections
4165 independent reflections
3758 reflections with *I* > 2σ(*I*)
*R*_int_ = 0.040

#### Refinement

*R*[*F* ^2^ > 2σ(*F* ^2^)] = 0.026
*wR*(*F* ^2^) = 0.064
*S* = 1.05
4165 reflections
370 parameters
42 restraints
H atoms treated by a mixture of independent and constrained refinement
Δρ_max_ = 1.81 e Å^−3^
Δρ_min_ = −0.46 e Å^−3^

### 

Data collection: *RAPID-AUTO* (Rigaku, 1998 ▶); cell refinement: *RAPID-AUTO*; data reduction: *CrystalClear* (Rigaku/MSC & Rigaku Corporation, 2002 ▶); program(s) used to solve structure: *SHELXS97* (Sheldrick, 2008 ▶); program(s) used to refine structure: *SHELXL97* (Sheldrick, 2008 ▶); molecular graphics: *X-SEED* (Barbour, 2001 ▶); software used to prepare material for publication: *publCIF* (Westrip, 2010 ▶).

## Supplementary Material

Crystal structure: contains datablock(s) global, I. DOI: 10.1107/S1600536811034398/qk2017sup1.cif

Structure factors: contains datablock(s) I. DOI: 10.1107/S1600536811034398/qk2017Isup2.hkl

Additional supplementary materials:  crystallographic information; 3D view; checkCIF report

## Figures and Tables

**Table 1:** Hydrogen-bond geometry (Å, °)

*D*—H⋯*A*	*D*—H	H⋯*A*	*D*⋯*A*	*D*—H⋯*A*
O1*W*—H11⋯O3*W*	0.82 (1)	1.90 (2)	2.671 (5)	157 (5)
O1*W*—H12⋯O6*W*^i^	0.82 (1)	1.85 (1)	2.677 (4)	177 (4)
O2*W*—H21⋯O4^ii^	0.82 (1)	1.89 (1)	2.705 (3)	172 (4)
O2*W*—H22⋯O5*W*^i^	0.82 (1)	1.88 (1)	2.694 (4)	169 (4)
O3*W*—H32⋯O5*W*	0.82 (1)	2.11 (6)	2.827 (5)	145 (9)
O4*W*—H41⋯O2	0.82 (1)	1.98 (2)	2.777 (4)	166 (5)
O4*W*—H42⋯O6^iii^	0.82 (1)	1.99 (2)	2.765 (4)	160 (5)
O5*W*—H51⋯O5^iii^	0.82 (1)	2.16 (1)	2.970 (4)	172 (5)
O5*W*—H52⋯N8^iv^	0.82 (1)	2.04 (1)	2.847 (5)	165 (4)
O6*W*—H61⋯O4*W*	0.82 (1)	2.02 (2)	2.824 (4)	168 (4)
O6*W*—H62⋯N2^v^	0.82 (1)	2.11 (2)	2.886 (4)	159 (5)
N3—H3*B*⋯O2	0.88	2.06	2.718 (5)	131
N6—H6*A*⋯O1^vi^	0.88	2.38	3.184 (4)	152
N6—H6*B*⋯O4	0.88	2.06	2.709 (4)	130
N9—H9*A*⋯O3^vii^	0.88	2.30	3.142 (4)	161
N9—H9*B*⋯O6	0.88	2.07	2.705 (5)	129

Symmetry codes: (i) 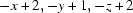; (ii) 
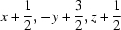
; (iii) 
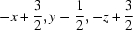
; (iv) 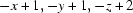; (v) 
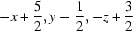
; (vi) 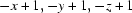; (vii) 
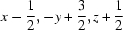
.

**Fig. 1.**
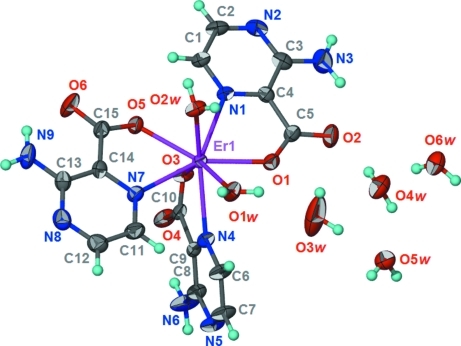
Thermal ellipsoid plot (Barbour, 2001) of Er(H2O)2(C5H4N3O2)3.4H2O at the 50% probability level; hydrogen toms are drawn as spheres of arbitrary radius.

**Fig. 2.**
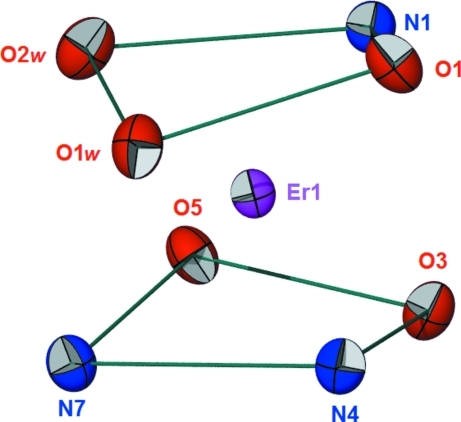
Square-antiprismatic coordination figure of Er in the title compound.

## supplementary crystallographic information

### Comment

The chelating ability of the 3-aminopyrazine-2-carboxylate anion is probably similar to that of the pyrazine-2-carboxylate anion, and the crystal structures of a number of lanthanum carboxylates have been reported. Hydrated lanthanum tris(pyrazine-2-carboxylate) adopts a chain motif (Leciejewicz *et al.*, 2004). The additional amino substitution in the 3-aminopyrazine-2-carboxylate should be expected to consolidate the crystal structure of the title erbium derivative through extensive hydrogen bonding. The water-coordinated Er^III^ atom in Er(H_2_O)_2_(C_5_H_4_N_3_O_2_)_3_^.^4H_2_O (Scheme I, Fig. 1) is *N*,*O*-chelated by three 3-aminopyrazine-2-carboxylate ions and has a square-antiprismatic coordination geometry. The two planes O3-O5-N7-N4 and O1-N1-O2W-O1W (r.m.s. deviation from planarity 0.147 and 0.159 Å, respectively) are nearly parallel to each other, the dihedral angle between them being only 2.4 (1) ° (Fig. 2).

The mononuclear molecule interacts with the lattice water molecules to generate a three-dimensional hydrogen-bonded network (Table 1).

### Experimental

Erbium nitrate hexahydrate (1 mmol) was added to a hot aqueous solution of 3-aminopyrazine-2-carboxylic acid (3 mmol). The solution was allowed to evaporate slowly at room temperature, and colorless prismatic crystals were isolated after five days.

### Refinement

Carbon- and nitrogen-bound H-atoms were placed in calculated positions (C–H 0.93 Å, N–H 0.8 Å) were included in the refinement in the riding model approximation, with *U*_iso_(H) set to 1.2*U*_eq_(C,N). The water H-atoms were located in a difference Fourier map, and were refined with distance restraints of O—H 0.82±0.01 Å and H···H 1.373±0.01 Å; their temperature factors were tied to those of the O atoms by a factor of 1.5 times.

The anisotropic temperature factors of the four lattice water molecules were restrained to be nearly isotropic (by using a 'tight' ISOR 0.01 restraint in *SHELXL*-97); this command had the effect of merely reducing the strong anisotropy of O3w only marginally.

The hydrogen positions H31 and H32 of O3w are questionable because this oxygen is obviously disordered showing principal mean square atomic displacements U of 0.261, 0.076, and 0.041 Å with the largest component directed to H31.

The final difference Fourier map had two peaks of 1.7, 1.8 *e*Å^-3^ in the vicinity of Er1 that are *trans* to each other are attributed to absorption effects.

### Crystal data

**Table:**

[Er(C_5_H_4_N_3_O_2_)_3_(H_2_O)_2_]·4H_2_O	*F*(000) = 1364
*M**_r_* = 689.69	*D*_x_ = 1.932 Mg m^−^^3^
Monoclinic, *P*2_1_/*n*	Mo *K*α radiation, λ = 0.71073 Å
Hall symbol: -P 2yn	Cell parameters from 17031 reflections
*a* = 9.1915 (9) Å	θ = 3.1–27.4°
*b* = 19.7152 (14) Å	µ = 3.62 mm^−^^1^
*c* = 13.5637 (11) Å	*T* = 291 K
β = 105.276 (3)°	Prism, colorless
*V* = 2371.1 (3) Å^3^	0.25 × 0.20 × 0.15 mm
*Z* = 4	

### Data collection

**Table:**

Rigaku R-AXIS RAPID IP diffractometer	4165 independent reflections
Radiation source: fine-focus sealed tube	3758 reflections with *I* > 2σ(*I*)
graphite	*R*_int_ = 0.040
ω scan	θ_max_ = 25.0°, θ_min_ = 3.1°
Absorption correction: multi-scan (*ABSCOR*; Higashi, 1995)	*h* = −10→10
*T*_min_ = 0.465, *T*_max_ = 0.613	*k* = −23→23
18438 measured reflections	*l* = −15→16

### Refinement

**Table:**

Refinement on *F*^2^	Primary atom site location: structure-invariant direct methods
Least-squares matrix: full	Secondary atom site location: difference Fourier map
*R*[*F*^2^ > 2σ(*F*^2^)] = 0.026	Hydrogen site location: inferred from neighbouring sites
*wR*(*F*^2^) = 0.064	H atoms treated by a mixture of independent and constrained refinement
*S* = 1.05	*w* = 1/[σ^2^(*F*_o_^2^) + (0.0371*P*)^2^ + 1.0656*P*] where *P* = (*F*_o_^2^ + 2*F*_c_^2^)/3
4165 reflections	(Δ/σ)_max_ = 0.001
370 parameters	Δρ_max_ = 1.81 e Å^−^^3^
42 restraints	Δρ_min_ = −0.46 e Å^−^^3^

### Special details

**Table:**

Geometry. All e.s.d.'s (except the e.s.d. in the dihedral angle between two l.s. planes) are estimated using the full covariance matrix. The cell e.s.d.'s are taken into account individually in the estimation of e.s.d.'s in distances, angles and torsion angles; correlations between e.s.d.'s in cell parameters are only used when they are defined by crystal symmetry. An approximate (isotropic) treatment of cell e.s.d.'s is used for estimating e.s.d.'s involving l.s. planes.

### Fractional atomic coordinates and isotropic or equivalent isotropic displacement parameters (Å^2^)

**Table:**

	*x*	*y*	*z*	*U*_iso_*/*U*_eq_	
Er1	0.642510 (16)	0.659798 (7)	0.765119 (11)	0.02540 (8)	
O1	0.8299 (3)	0.60156 (12)	0.72083 (19)	0.0371 (6)	
O2	1.0305 (3)	0.59246 (15)	0.6586 (2)	0.0541 (8)	
O3	0.5298 (3)	0.67906 (12)	0.59543 (18)	0.0356 (6)	
O4	0.3591 (4)	0.65762 (11)	0.4484 (2)	0.0436 (7)	
O5	0.5324 (3)	0.76501 (12)	0.76143 (19)	0.0351 (6)	
O6	0.4034 (4)	0.84026 (12)	0.8270 (3)	0.0607 (10)	
O1W	0.7027 (3)	0.57364 (13)	0.88614 (19)	0.0384 (6)	
H11	0.776 (3)	0.550 (2)	0.888 (3)	0.058*	
H12	0.689 (4)	0.574 (2)	0.9438 (15)	0.058*	
O2W	0.7686 (3)	0.71168 (12)	0.9192 (2)	0.0402 (6)	
H21	0.796 (4)	0.7516 (8)	0.922 (3)	0.060*	
H22	0.834 (4)	0.6904 (16)	0.961 (3)	0.060*	
O3W	0.8829 (6)	0.4707 (3)	0.8682 (5)	0.126 (2)	
H31	0.949 (8)	0.492 (4)	0.909 (6)	0.189*	
H32	0.917 (9)	0.433 (2)	0.860 (7)	0.189*	
O4W	1.0911 (4)	0.46790 (16)	0.7564 (2)	0.0605 (8)	
H41	1.082 (7)	0.5021 (13)	0.721 (3)	0.091*	
H42	1.077 (7)	0.4345 (14)	0.720 (3)	0.091*	
O5W	1.0060 (4)	0.34165 (13)	0.9322 (3)	0.0468 (7)	
H51	0.996 (5)	0.317 (2)	0.882 (2)	0.070*	
H52	0.948 (5)	0.329 (2)	0.965 (3)	0.070*	
O6W	1.3302 (4)	0.42757 (15)	0.9235 (2)	0.0507 (7)	
H61	1.257 (4)	0.443 (2)	0.881 (3)	0.076*	
H62	1.348 (5)	0.3897 (12)	0.906 (3)	0.076*	
N1	0.8318 (3)	0.73453 (14)	0.7137 (2)	0.0303 (7)	
N2	1.0252 (4)	0.80723 (17)	0.6265 (3)	0.0449 (8)	
N3	1.1379 (5)	0.7086 (2)	0.5957 (3)	0.0651 (11)	
H3A	1.1979	0.7327	0.5684	0.078*	
H3B	1.1463	0.6641	0.5985	0.078*	
N4	0.4864 (3)	0.56156 (14)	0.6733 (2)	0.0318 (7)	
N5	0.3145 (4)	0.45871 (16)	0.5564 (3)	0.0486 (9)	
N6	0.2459 (5)	0.53012 (18)	0.4193 (3)	0.0630 (12)	
H6A	0.1937	0.4964	0.3846	0.076*	
H6B	0.2476	0.5698	0.3898	0.076*	
N7	0.4190 (4)	0.66166 (13)	0.8414 (2)	0.0335 (7)	
N8	0.1896 (4)	0.67860 (19)	0.9389 (3)	0.0453 (8)	
N9	0.2151 (4)	0.79374 (18)	0.9351 (3)	0.0540 (9)	
H9A	0.1454	0.7968	0.9686	0.065*	
H9B	0.2564	0.8307	0.9181	0.065*	
C1	0.8279 (4)	0.80226 (18)	0.7093 (3)	0.0363 (8)	
H1	0.7607	0.8262	0.7368	0.044*	
C2	0.9235 (5)	0.83638 (19)	0.6642 (3)	0.0446 (11)	
H2	0.9155	0.8834	0.6601	0.054*	
C3	1.0340 (4)	0.7394 (2)	0.6325 (3)	0.0408 (9)	
C4	0.9350 (4)	0.70231 (18)	0.6775 (3)	0.0304 (8)	
C5	0.9342 (4)	0.62603 (18)	0.6853 (3)	0.0343 (8)	
C6	0.4753 (5)	0.50025 (18)	0.7126 (3)	0.0426 (10)	
H6	0.5259	0.4911	0.7801	0.051*	
C7	0.3889 (5)	0.45048 (19)	0.6531 (3)	0.0492 (11)	
H7	0.3827	0.4085	0.6831	0.059*	
C8	0.3228 (4)	0.52110 (18)	0.5168 (3)	0.0376 (9)	
C9	0.4114 (4)	0.57249 (16)	0.5762 (3)	0.0278 (7)	
C10	0.4334 (4)	0.64121 (16)	0.5359 (3)	0.0289 (8)	
C11	0.3538 (5)	0.6086 (2)	0.8741 (3)	0.0448 (10)	
H11A	0.3873	0.5649	0.8662	0.054*	
C12	0.2380 (5)	0.6179 (2)	0.9193 (3)	0.0485 (10)	
H12A	0.1910	0.5798	0.9371	0.058*	
C13	0.2584 (4)	0.7329 (2)	0.9100 (3)	0.0373 (9)	
C14	0.3703 (4)	0.72401 (18)	0.8572 (3)	0.0317 (8)	
C15	0.4411 (4)	0.78162 (17)	0.8129 (3)	0.0353 (8)	

### Atomic displacement parameters (Å^2^)

**Table:**

	*U*^11^	*U*^22^	*U*^33^	*U*^12^	*U*^13^	*U*^23^
Er1	0.03025 (11)	0.02031 (10)	0.02724 (11)	−0.00154 (5)	0.01038 (7)	−0.00073 (6)
O1	0.0411 (15)	0.0264 (13)	0.0501 (15)	−0.0003 (11)	0.0230 (12)	0.0004 (11)
O2	0.0544 (19)	0.0451 (16)	0.075 (2)	0.0148 (14)	0.0381 (16)	0.0058 (15)
O3	0.0485 (16)	0.0273 (12)	0.0298 (13)	−0.0096 (11)	0.0084 (12)	0.0006 (11)
O4	0.0597 (19)	0.0303 (14)	0.0338 (15)	0.0010 (11)	0.0001 (14)	0.0052 (11)
O5	0.0392 (14)	0.0290 (12)	0.0426 (14)	0.0041 (10)	0.0204 (12)	0.0038 (11)
O6	0.090 (3)	0.0277 (15)	0.083 (3)	0.0159 (14)	0.054 (2)	0.0077 (13)
O1W	0.0508 (17)	0.0329 (14)	0.0324 (13)	0.0075 (12)	0.0125 (12)	0.0027 (12)
O2W	0.0534 (17)	0.0269 (13)	0.0368 (14)	−0.0042 (12)	0.0055 (12)	−0.0026 (12)
O3W	0.161 (5)	0.089 (3)	0.167 (5)	0.076 (3)	0.112 (4)	0.060 (3)
O4W	0.073 (2)	0.0457 (17)	0.0593 (19)	0.0137 (17)	0.0118 (16)	−0.0056 (15)
O5W	0.0510 (19)	0.0420 (17)	0.0481 (18)	−0.0026 (12)	0.0139 (14)	−0.0021 (12)
O6W	0.062 (2)	0.0480 (17)	0.0440 (16)	0.0136 (14)	0.0177 (14)	−0.0002 (13)
N1	0.0354 (17)	0.0245 (15)	0.0313 (15)	−0.0040 (12)	0.0093 (13)	−0.0007 (12)
N2	0.045 (2)	0.046 (2)	0.0457 (19)	−0.0139 (16)	0.0156 (16)	0.0032 (16)
N3	0.063 (3)	0.059 (2)	0.093 (3)	0.004 (2)	0.056 (2)	0.013 (2)
N4	0.0395 (17)	0.0255 (14)	0.0312 (16)	−0.0006 (12)	0.0104 (13)	0.0018 (12)
N5	0.062 (2)	0.0291 (16)	0.046 (2)	−0.0116 (15)	−0.0006 (17)	0.0005 (15)
N6	0.089 (3)	0.0351 (19)	0.046 (2)	−0.0175 (19)	−0.016 (2)	0.0035 (17)
N7	0.0396 (18)	0.0274 (16)	0.0365 (17)	−0.0002 (12)	0.0152 (14)	−0.0021 (12)
N8	0.043 (2)	0.052 (2)	0.046 (2)	0.0038 (17)	0.0209 (16)	0.0047 (17)
N9	0.064 (2)	0.047 (2)	0.065 (2)	0.0225 (18)	0.0408 (19)	0.0104 (18)
C1	0.044 (2)	0.0271 (18)	0.0381 (19)	−0.0052 (16)	0.0121 (17)	−0.0041 (17)
C2	0.055 (3)	0.032 (2)	0.046 (2)	−0.0138 (17)	0.011 (2)	−0.0011 (17)
C3	0.036 (2)	0.047 (2)	0.039 (2)	−0.0071 (17)	0.0077 (17)	0.0029 (18)
C4	0.0292 (18)	0.0337 (18)	0.0287 (17)	−0.0017 (15)	0.0080 (14)	0.0017 (15)
C5	0.039 (2)	0.0290 (19)	0.036 (2)	0.0013 (16)	0.0126 (17)	−0.0017 (16)
C6	0.061 (3)	0.0252 (18)	0.036 (2)	−0.0073 (17)	0.0026 (18)	0.0031 (16)
C7	0.068 (3)	0.0248 (19)	0.047 (2)	−0.0118 (18)	0.002 (2)	0.0062 (18)
C8	0.044 (2)	0.0288 (18)	0.0362 (19)	−0.0022 (16)	0.0033 (16)	0.0009 (16)
C9	0.0305 (18)	0.0230 (16)	0.0304 (18)	0.0019 (13)	0.0091 (14)	−0.0012 (14)
C10	0.036 (2)	0.0215 (16)	0.0329 (19)	0.0043 (15)	0.0144 (16)	0.0015 (15)
C11	0.055 (3)	0.033 (2)	0.054 (2)	−0.0084 (18)	0.028 (2)	−0.0024 (19)
C12	0.049 (3)	0.048 (2)	0.055 (3)	−0.008 (2)	0.025 (2)	−0.001 (2)
C13	0.038 (2)	0.043 (2)	0.0329 (19)	0.0078 (17)	0.0114 (16)	0.0031 (17)
C14	0.0317 (19)	0.0324 (18)	0.0307 (18)	0.0058 (15)	0.0078 (15)	0.0023 (15)
C15	0.037 (2)	0.0300 (19)	0.039 (2)	0.0052 (16)	0.0106 (17)	0.0042 (16)

### Geometric parameters (Å, °)

**Table:**

Er1—O1	2.278 (2)	N3—H3A	0.8800
Er1—O3	2.293 (2)	N3—H3B	0.8800
Er1—O5	2.303 (2)	N4—C9	1.334 (4)
Er1—O1W	2.325 (2)	N4—C6	1.336 (4)
Er1—O2W	2.341 (2)	N5—C7	1.319 (5)
Er1—N1	2.515 (3)	N5—C8	1.353 (5)
Er1—N7	2.532 (3)	N6—C8	1.337 (5)
Er1—N4	2.534 (3)	N6—H6A	0.8800
O1—C5	1.275 (4)	N6—H6B	0.8800
O2—C5	1.234 (5)	N7—C11	1.339 (5)
O3—C10	1.272 (4)	N7—C14	1.344 (4)
O4—C10	1.246 (4)	N8—C12	1.327 (6)
O5—C15	1.268 (4)	N8—C13	1.353 (5)
O6—C15	1.236 (4)	N9—C13	1.336 (5)
O1W—H11	0.82 (1)	N9—H9A	0.8800
O1W—H12	0.82 (1)	N9—H9B	0.8800
O2W—H21	0.82 (1)	C1—C2	1.372 (6)
O2W—H22	0.82 (1)	C1—H1	0.9300
O3W—H31	0.82 (1)	C2—H2	0.9300
O3W—H32	0.82 (1)	C3—C4	1.423 (5)
O4W—H41	0.82 (1)	C4—C5	1.508 (5)
O4W—H42	0.82 (1)	C6—C7	1.381 (5)
O5W—H51	0.82 (1)	C6—H6	0.9300
O5W—H52	0.82 (1)	C7—H7	0.9300
O6W—H61	0.82 (1)	C8—C9	1.411 (5)
O6W—H62	0.82 (1)	C9—C10	1.495 (5)
N1—C1	1.337 (5)	C11—C12	1.374 (6)
N1—C4	1.338 (5)	C11—H11A	0.9300
N2—C2	1.311 (6)	C12—H12A	0.9300
N2—C3	1.341 (5)	C13—C14	1.411 (5)
N3—C3	1.335 (5)	C14—C15	1.510 (5)
			
O1—Er1—O3	89.52 (9)	H6A—N6—H6B	120.0
O1—Er1—O5	143.77 (9)	C11—N7—C14	117.8 (3)
O3—Er1—O5	75.48 (9)	C11—N7—Er1	127.3 (3)
O1—Er1—O1W	76.25 (9)	C14—N7—Er1	114.7 (2)
O3—Er1—O1W	142.19 (9)	C12—N8—C13	116.6 (4)
O5—Er1—O1W	134.01 (9)	C13—N9—H9A	120.0
O1—Er1—O2W	102.99 (10)	C13—N9—H9B	120.0
O3—Er1—O2W	144.11 (8)	H9A—N9—H9B	120.0
O5—Er1—O2W	74.76 (9)	N1—C1—C2	119.8 (4)
O1W—Er1—O2W	73.69 (9)	N1—C1—H1	120.1
O1—Er1—N1	66.16 (9)	C2—C1—H1	120.1
O3—Er1—N1	77.71 (9)	N2—C2—C1	124.4 (4)
O5—Er1—N1	78.37 (9)	N2—C2—H2	117.8
O1W—Er1—N1	124.79 (10)	C1—C2—H2	117.8
O2W—Er1—N1	77.04 (10)	N3—C3—N2	117.9 (4)
O1—Er1—N7	150.00 (9)	N3—C3—C4	121.9 (4)
O3—Er1—N7	101.96 (10)	N2—C3—C4	120.2 (4)
O5—Er1—N7	66.21 (8)	N1—C4—C3	120.6 (3)
O1W—Er1—N7	77.86 (10)	N1—C4—C5	115.5 (3)
O2W—Er1—N7	83.85 (10)	C3—C4—C5	123.9 (3)
N1—Er1—N7	143.12 (9)	O2—C5—O1	125.2 (3)
O1—Er1—N4	81.64 (9)	O2—C5—C4	119.8 (3)
O3—Er1—N4	65.52 (9)	O1—C5—C4	115.0 (3)
O5—Er1—N4	119.29 (9)	N4—C6—C7	120.2 (3)
O1W—Er1—N4	77.71 (9)	N4—C6—H6	119.9
O2W—Er1—N4	148.85 (9)	C7—C6—H6	119.9
N1—Er1—N4	130.93 (9)	N5—C7—C6	124.1 (3)
N7—Er1—N4	78.27 (9)	N5—C7—H7	117.9
C5—O1—Er1	127.2 (2)	C6—C7—H7	117.9
C10—O3—Er1	126.5 (2)	N6—C8—N5	116.3 (3)
C15—O5—Er1	124.9 (2)	N6—C8—C9	122.9 (3)
Er1—O1W—H11	119 (3)	N5—C8—C9	120.7 (3)
Er1—O1W—H12	127 (3)	N4—C9—C8	121.2 (3)
H11—O1W—H12	108 (4)	N4—C9—C10	115.0 (3)
Er1—O2W—H21	122 (3)	C8—C9—C10	123.7 (3)
Er1—O2W—H22	120 (3)	O4—C10—O3	124.6 (3)
H21—O2W—H22	107 (5)	O4—C10—C9	119.4 (3)
H31—O3W—H32	108 (4)	O3—C10—C9	116.0 (3)
H41—O4W—H42	110 (4)	N7—C11—C12	120.6 (4)
H51—O5W—H52	109 (4)	N7—C11—H11A	119.7
H61—O6W—H62	109 (4)	C12—C11—H11A	119.7
C1—N1—C4	118.1 (3)	N8—C12—C11	123.4 (4)
C1—N1—Er1	125.8 (2)	N8—C12—H12A	118.3
C4—N1—Er1	115.7 (2)	C11—C12—H12A	118.3
C2—N2—C3	116.8 (3)	N9—C13—N8	116.3 (4)
C3—N3—H3A	120.0	N9—C13—C14	123.1 (4)
C3—N3—H3B	120.0	N8—C13—C14	120.6 (3)
H3A—N3—H3B	120.0	N7—C14—C13	120.8 (3)
C9—N4—C6	117.8 (3)	N7—C14—C15	115.4 (3)
C9—N4—Er1	116.1 (2)	C13—C14—C15	123.8 (3)
C6—N4—Er1	126.1 (2)	O6—C15—O5	125.5 (4)
C7—N5—C8	116.0 (3)	O6—C15—C14	118.3 (4)
C8—N6—H6A	120.0	O5—C15—C14	116.2 (3)
C8—N6—H6B	120.0		
			
O3—Er1—O1—C5	71.6 (3)	N4—Er1—N7—C14	−141.2 (3)
O5—Er1—O1—C5	7.5 (4)	C4—N1—C1—C2	3.0 (5)
O1W—Er1—O1—C5	−143.7 (3)	Er1—N1—C1—C2	−169.2 (3)
O2W—Er1—O1—C5	−74.4 (3)	C3—N2—C2—C1	0.5 (6)
N1—Er1—O1—C5	−5.1 (3)	N1—C1—C2—N2	−2.3 (6)
N7—Er1—O1—C5	−174.8 (3)	C2—N2—C3—N3	−179.3 (4)
N4—Er1—O1—C5	137.0 (3)	C2—N2—C3—C4	0.5 (5)
O1—Er1—O3—C10	89.3 (3)	C1—N1—C4—C3	−2.1 (5)
O5—Er1—O3—C10	−124.0 (3)	Er1—N1—C4—C3	171.0 (3)
O1W—Er1—O3—C10	22.8 (4)	C1—N1—C4—C5	179.6 (3)
O2W—Er1—O3—C10	−158.8 (3)	Er1—N1—C4—C5	−7.4 (3)
N1—Er1—O3—C10	155.0 (3)	N3—C3—C4—N1	−179.9 (4)
N7—Er1—O3—C10	−62.7 (3)	N2—C3—C4—N1	0.3 (5)
N4—Er1—O3—C10	8.3 (3)	N3—C3—C4—C5	−1.7 (6)
O1—Er1—O5—C15	−165.9 (3)	N2—C3—C4—C5	178.5 (3)
O3—Er1—O5—C15	125.7 (3)	Er1—O1—C5—O2	−177.1 (3)
O1W—Er1—O5—C15	−26.5 (3)	Er1—O1—C5—C4	3.2 (4)
O2W—Er1—O5—C15	−74.6 (3)	N1—C4—C5—O2	−176.3 (4)
N1—Er1—O5—C15	−154.1 (3)	C3—C4—C5—O2	5.5 (5)
N7—Er1—O5—C15	15.4 (3)	N1—C4—C5—O1	3.4 (4)
N4—Er1—O5—C15	75.2 (3)	C3—C4—C5—O1	−174.8 (3)
O1—Er1—N1—C1	178.8 (3)	C9—N4—C6—C7	0.3 (6)
O3—Er1—N1—C1	83.8 (3)	Er1—N4—C6—C7	−178.3 (3)
O5—Er1—N1—C1	6.4 (3)	C8—N5—C7—C6	−2.0 (7)
O1W—Er1—N1—C1	−129.7 (3)	N4—C6—C7—N5	0.7 (7)
O2W—Er1—N1—C1	−70.5 (3)	C7—N5—C8—N6	−179.1 (4)
N7—Er1—N1—C1	−9.8 (4)	C7—N5—C8—C9	2.3 (6)
N4—Er1—N1—C1	125.2 (3)	C6—N4—C9—C8	0.1 (5)
O1—Er1—N1—C4	6.4 (2)	Er1—N4—C9—C8	178.9 (3)
O3—Er1—N1—C4	−88.6 (2)	C6—N4—C9—C10	−177.7 (3)
O5—Er1—N1—C4	−166.1 (2)	Er1—N4—C9—C10	1.0 (4)
O1W—Er1—N1—C4	57.8 (3)	N6—C8—C9—N4	180.0 (4)
O2W—Er1—N1—C4	117.1 (2)	N5—C8—C9—N4	−1.5 (6)
N7—Er1—N1—C4	177.8 (2)	N6—C8—C9—C10	−2.3 (6)
N4—Er1—N1—C4	−47.2 (3)	N5—C8—C9—C10	176.2 (4)
O1—Er1—N4—C9	−97.5 (3)	Er1—O3—C10—O4	169.5 (3)
O3—Er1—N4—C9	−4.2 (2)	Er1—O3—C10—C9	−10.8 (4)
O5—Er1—N4—C9	51.0 (3)	N4—C9—C10—O4	−174.8 (3)
O1W—Er1—N4—C9	−175.1 (3)	C8—C9—C10—O4	7.4 (5)
O2W—Er1—N4—C9	161.2 (2)	N4—C9—C10—O3	5.5 (5)
N1—Er1—N4—C9	−49.4 (3)	C8—C9—C10—O3	−172.3 (3)
N7—Er1—N4—C9	104.9 (3)	C14—N7—C11—C12	2.0 (6)
O1—Er1—N4—C6	81.2 (3)	Er1—N7—C11—C12	176.3 (3)
O3—Er1—N4—C6	174.5 (3)	C13—N8—C12—C11	1.4 (6)
O5—Er1—N4—C6	−130.4 (3)	N7—C11—C12—N8	−4.1 (7)
O1W—Er1—N4—C6	3.5 (3)	C12—N8—C13—N9	−176.4 (4)
O2W—Er1—N4—C6	−20.1 (4)	C12—N8—C13—C14	3.1 (6)
N1—Er1—N4—C6	129.2 (3)	C11—N7—C14—C13	2.4 (5)
N7—Er1—N4—C6	−76.4 (3)	Er1—N7—C14—C13	−172.6 (3)
O1—Er1—N7—C11	−4.6 (5)	C11—N7—C14—C15	−176.4 (3)
O3—Er1—N7—C11	105.9 (3)	Er1—N7—C14—C15	8.6 (4)
O5—Er1—N7—C11	174.0 (4)	N9—C13—C14—N7	174.3 (4)
O1W—Er1—N7—C11	−35.4 (3)	N8—C13—C14—N7	−5.1 (5)
O2W—Er1—N7—C11	−110.0 (3)	N9—C13—C14—C15	−7.0 (6)
N1—Er1—N7—C11	−168.7 (3)	N8—C13—C14—C15	173.6 (3)
N4—Er1—N7—C11	44.4 (3)	Er1—O5—C15—O6	165.5 (3)
O1—Er1—N7—C14	169.9 (2)	Er1—O5—C15—C14	−16.6 (4)
O3—Er1—N7—C14	−79.6 (3)	N7—C14—C15—O6	−178.3 (4)
O5—Er1—N7—C14	−11.6 (2)	C13—C14—C15—O6	2.9 (6)
O1W—Er1—N7—C14	139.1 (3)	N7—C14—C15—O5	3.6 (5)
O2W—Er1—N7—C14	64.5 (3)	C13—C14—C15—O5	−175.2 (3)
N1—Er1—N7—C14	5.8 (3)		

### Hydrogen-bond geometry (Å, °)

**Table:**

*D*—H···*A*	*D*—H	H···*A*	*D*···*A*	*D*—H···*A*
O1W—H11···O3W	0.82 (1)	1.90 (2)	2.671 (5)	157 (5)
O1W—H12···O6W^i^	0.82 (1)	1.85 (1)	2.677 (4)	177 (4)
O2W—H21···O4^ii^	0.82 (1)	1.89 (1)	2.705 (3)	172 (4)
O2W—H22···O5W^i^	0.82 (1)	1.88 (1)	2.694 (4)	169 (4)
O3W—H32···O5W	0.82 (1)	2.11 (6)	2.827 (5)	145 (9)
O4W—H41···O2	0.82 (1)	1.98 (2)	2.777 (4)	166 (5)
O4W—H42···O6^iii^	0.82 (1)	1.99 (2)	2.765 (4)	160 (5)
O5W—H51···O5^iii^	0.82 (1)	2.16 (1)	2.970 (4)	172 (5)
O5W—H52···N8^iv^	0.82 (1)	2.04 (1)	2.847 (5)	165 (4)
O6W—H61···O4W	0.82 (1)	2.02 (2)	2.824 (4)	168 (4)
O6W—H62···N2^v^	0.82 (1)	2.11 (2)	2.886 (4)	159 (5)
N3—H3B···O2	0.88	2.06	2.718 (5)	131
N6—H6A···O1^vi^	0.88	2.38	3.184 (4)	152
N6—H6B···O4	0.88	2.06	2.709 (4)	130
N9—H9A···O3^vii^	0.88	2.30	3.142 (4)	161
N9—H9B···O6	0.88	2.07	2.705 (5)	129

Symmetry codes: (i) −*x*+2, −*y*+1, −*z*+2; (ii) *x*+1/2, −*y*+3/2, *z*+1/2; (iii) −*x*+3/2, *y*−1/2, −*z*+3/2; (iv) −*x*+1, −*y*+1, −*z*+2; (v) −*x*+5/2, *y*−1/2, −*z*+3/2; (vi) −*x*+1, −*y*+1, −*z*+1; (vii) *x*−1/2, −*y*+3/2, *z*+1/2.

## References

[bb1] Barbour, L. J. (2001). *J. Supramol. Chem.* **1**, 189–191.

[bb2] Higashi, T. (1995). *ABSCOR* Rigaku Corporation, Tokyo, Japan.

[bb3] Leciejewicz, J., Ptasiewicz-Bak, H., Premkumar, T. & Govindarajan, S. (2004). *J. Coord. Chem.* **57**, 97–103.

[bb4] Rigaku (1998). *RAPID-AUTO* Rigaku Corporation, Tokyo, Japan.

[bb5] Rigaku/MSC & Rigaku Corporation (2002). *CrystalClear* Rigaku/MSC Inc., The Woodlands, Texas, USA.

[bb6] Sheldrick, G. M. (2008). *Acta Cryst.* A**64**, 112–122. 10.1107/S0108767307043930 18156677

[bb7] Westrip, S. P. (2010). *J. Appl. Cryst.* **43**, 920–925.

